# Human-Related Factors Regulate the Spatial Ecology of Domestic Cats in Sensitive Areas for Conservation

**DOI:** 10.1371/journal.pone.0025970

**Published:** 2011-10-17

**Authors:** Joaquim P. Ferreira, Inês Leitão, Margarida Santos-Reis, Eloy Revilla

**Affiliations:** 1 Departamento de Biologia Animal, Faculdade de Ciências de Lisboa, Centro de Biologia Ambiental, Universidade de Lisboa, Lisboa, Portugal; 2 Department of Conservation Biology, Estación Biológica de Doñana Consejo Superior de Investigaciones Científicas (CSIC), Seville, Spain; 3 Departamento de Biologia and Centre for Environmental and Marine Studies (CESAM), Universidade de Aveiro Campus Universitário de Santiago, Aveiro, Portugal; Institut Pluridisciplinaire Hubert Curien, France

## Abstract

**Background:**

Domestic cats ranging freely in natural areas are a conservation concern due to competition, predation, disease transmission or hybridization with wildcats. In order to improve our ability to design effective control policies, we investigate the factors affecting their numbers and space use in natural areas of continental Europe.

**Methodology/Principal Findings:**

We describe the patterns of cat presence, abundance and space use and analyse the associated environmental and human constraints in a well-preserved Mediterranean natural area with small scattered local farms. We failed in detecting cats in areas away from human settlements (trapping effort above 4000 trap-nights), while we captured 30 individuals near inhabited farms. We identified 130 cats, all of them in farms still in use by people (30% of 128 farms). All cats were free-ranging and very wary of people. The main factor explaining the presence of cats was the presence of people, while the number of cats per farm was mostly affected by the occasional food provisioning with human refuse and the presence of people. The home ranges of eight radio tagged cats were centred at inhabited farms. Males went furthest away from the farms during the mating season (3.8 km on average, maximum 6.3 km), using inhabited farms as stepping-stones in their mating displacements (2.2 km of maximum inter-farm distance moved). In their daily movements, cats notably avoided entering in areas with high fox density.

**Conclusions:**

The presence, abundance and space use of cats were heavily dependent on human settlements. Any strategy aiming at reducing their impact in areas of conservation concern should aim at the presence of settlements and their spatial spread and avoid any access to human refuse. The movements of domestic cats would be limited in areas with large patches of natural vegetation providing good conditions for other carnivore mammals such as red foxes.

## Introduction

The presence of domestic species in the wild often represents a conservation problem due to competition with and predation of wild species and due to the potential hybridization with the wild ancestor types [1]–[4]. Moreover, domestic species act as reservoirs for many diseases [1]. The canine distemper epidemics transmitted by domestic dogs in the Serengeti lion *Panthera leo* population, caused a mortality of 30% of the individuals [5], and the decline of African wild dogs *Lycaon picus*, in the Serengeti due to the same disease [6]. The domestic cat (*Felis catus*) is no exception and is currently considered a major conservation problem. It is the carnivore species with a wider distribution range, being present in all continents and in many islands, including several subantartic islands [2], [7], [8].

The effects of domestic cats on wildlife depend on where they are found and on the factors controlling their numbers and space use [9]. House cats are present in large numbers in urban and suburban areas around the globe, where, due to their high numbers, they can have a substantial impact on wildlife, even when they do not need to hunt to survive.

However, cats whose needs are not satisfied by people (at least not intentionally) pose the main conservation threats. Domestic cats may live and reproduce with little (in the case of stray cats) or no human intervention (feral cats) and survive by scavenging or hunting. The almost unlimited food supplies that cities provide allow for the presence of large numbers of feral cats; for example, about 30 million cats are estimated to live in the streets of the United States [10]. The number of feral cats follows the gradient of availability of human-related food resources and refuge from urban and suburban areas to rural areas, where the availability is much lower than in cities [11]. Free-ranging domestic cats live close to human settlements, and their home-range size varies with human density and with food availability and distribution [12]. At relatively low densities (less than 10 cats per km^2^), as in low humanized natural-rural areas, ranges are large and the rate of intra-specific encounters is low [13]. Size and stability of domestic cat populations depend therefore on a local combination of favorable environmental conditions providing food and refuge. Within this gradient, domestic cat populations exhibit varying degrees of dependence on humans, including feral cats living and reproducing freely at low densities in well-preserved natural areas [3], [14], [15]. The most problematic individuals are those living or expending time in natural areas, where they have access to rare or endangered prey, may get in contact with wild endangered carnivores and may interbreed with European wildcats (*Felis silvestris*). In many island ecosystems domestic cats are dominant predators that cause a very relevant impact on breeding seabird colonies and endemic species [16], [17], [18]. In mainland areas, house cats also have a record as a subsidised exotic predator of native species [19], [20]. In addition to their predatory impact, domestic cats act as reservoirs in the transmission of numerous diseases to other species [21], [22]. In the case of the Iberian lynx (*Lynx pardinus*), the most endangered feline of the world, the transmission of Feline Immunodeficiency Virus (FIV) and Feline Leukemia Virus (FeLV) by domestic cats may become a serious threat to their populations [23], [24]. Another major problem of free ranging domestic cats is introgressive hybridization with European wildcats [25]–[27]. Extensive hybridization has been described in Hungary and Scotland, contrasting with occasional interbreeding in Italy, France and Germany [28]–[30]. Much of the hybridizations probably occurred in areas where the extension of the spatial overlap between the two species is higher, especially when wildcat populations are already at low densities [31]. It is therefore important to understand what may affect the distribution of domestic cats and how they move in sensitive areas for conservation in order to minimize contact with species such as the European wild cat. In this context we investigate the factors associated with the presence, abundance and space use by free-ranging domestic cats in a well-preserved natural area with very low human density distributed in isolated farm settlements. The area is representative of well-preserved Mediterranean habitats where an Iberian lynx reintroduction program is planned [32] and where European wildcats could persist [33]. We aim at describing the patterns of occurrence and abundance of domestic cats, as well as space use (e.g. home range, movements and habitat use) and the associated environmental and human constraints that could influence these patterns. A priori, we expected that free-ranging domestic cats would be heavily dependent on human-related descriptors for individuals inhabiting near human settlements. By contrast, environmental features, such as those describing the availability of food and/or protection, should become much more relevant for cats living farther away. Information on those human and environmental determinants should prove useful when managing populations of domestic cats in sensitive natural areas.

## Methods

### Study area

**Figure 1:**
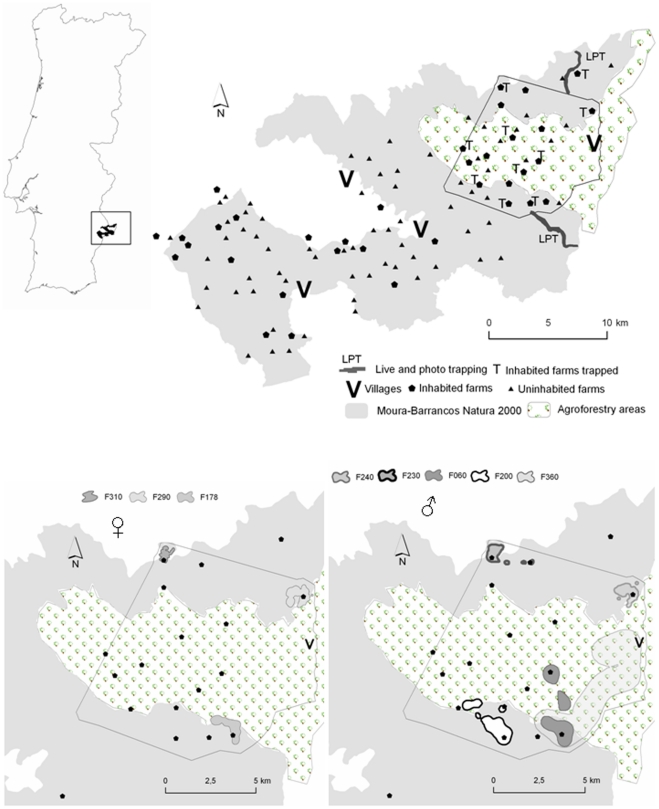
Study area defined by the Moura-Barrancos Natura 2000 site and the surrounding agroforestry area. The outline represents the minimum convex polygon encompassing all radiolocations of marked cats, and is presented below in detail to show the home ranges of the domestic cats (95% Kernel isolines) of males (right) and females (left).

The study was carried out in Moura-Barrancos Nature 2000 site (43,309 ha) and part of a Bird Special Protection Area, encompassing the agroforestry areas around the village of Barrancos in the Southeast Portuguese-Spanish border (between 38°13′N - 37°57′N and 7°24′O - 6°59′O, Figure 1) [34]. This landscape is a typically well-preserved Mediterranean forested area, dominated by holm oak woodlands (*Quercus rotundifolia*), patches of sclerophylous scrubland and rocky areas and boulders along the main rivers and streams. Elevation ranges between 200 and 400 m. There are no villages within the Natura 2000 site and human settlements are reduced to isolated traditional farms. The climate is characterized by dry warm summers and cold winters. Human activity is spatially limited, and consists in cattle rising, traditional agriculture and game hunting. This agro-silvo-pastoral system is characterized by a heterogeneous combination of patches with open tree cover for cattle grazing (montado or dehesa) and shrubby forest patches. Moura-Barrancos Natura 2000 study site belongs to two municipalities: Barrancos, with a single village occupied by about 1,800 people (with a municipal density of 10.7 inhabitants/km^2^) and Moura that encompasses five small villages close to the study area (17.1 inhabitants/km^2^) [34]. The study area potentially offers suitable habitat for European wildcats and the gradient between no human occupations to isolated farms offers a landscape context where hybridization between European wildcats and free-ranging domestic cats might occur. The Natura 2000 site was created, among other reasons, because it was one of the last strongholds of the Iberian lynx in Portugal [32], [35].

### Live-and photo-trapping

In order to determine the presence and abundance of domestic cats and to obtain individuals to radiotag, we implemented a live-trapping program both in scrubland areas far from human settlements and at inhabited farms located within or at the edge of the Natura 2000 site (Figure 1). Additionally, we performed intensive photo-trapping campaigns in the same natural areas as live trapping.

### Live-trapping in scrubland

In scrubland areas, we selected two sites with well-preserved natural vegetation (Figure 1). Trapping occurred between February and July 2006 and March and July 2007 with 14 box-traps (model 608, Tomahawk Live Trap Co., Wisconsin, United States of America). We located the box-traps in protected places with thick vegetation or rocky cover, separated by between 300 and 500 m and in a range of 1.3 to 4.5 km to the nearest inhabited farm (Figure 1). We baited the traps with a live pigeon to maximise the capture probability of all carnivore species. The pigeon was fed daily and protected inside the trap to avoid being captured. The capture and handling of wild carnivores and domestic cats was implemented by qualified people according to Portuguese legislation. Accreditation and permissions were granted by the Instituto para a Conservação da Natureza e Biodiversidade de Portugal (ICNB) (license 729/07/DAC/DCGB). We made all efforts to minimize suffering of both carnivores and pigeons. We checked the traps daily after sunrise.

### Live-trapping in farms

The trapping campaign at farms occurred in January and May 2007 in 10 inhabited farms within our study area. We carried out the live trapping around households using five traps per farm. Traps were baited with fresh fish and were checked twice a day (sunrise and sunset). The inhabited farms selected for the captures of radio tagged domestic cats represent well preserved game areas (Figure 1): Noudar Castle (NC), which with 980 ha is located inside the Natural Park; Coutada Frades (CF) with 468 ha and Contenda Forest Area (CFA) with 5309 ha, and Russianas (RUS) with 1480 ha, both of which are only partially devoted to grazing.

### Manipulation of cats

The animals captured were weighted after being anaesthetized with an intra-muscular injection of medetomidine hydrochloride (Domitors®, Espoo, Finland; 0.1 mg mL^−1^) and ketamine hydrochloride (Imalgenes®, Lyon, France; 1 g mL^−1^) in a 2∶1 proportion. After handling, we reversed the anaesthesia with 0.08 mg/kg of Antisedan. We estimated the age class (young/adult) from body weight and dentition. We considered adults those animals with full adult dentition and a weight above 2.5 kg and 3.5 kg for females and males respectively [12]. In total we marked eight cats with radio-collars with activity sensors (Telonics model 105, Telonics Inc, Mesa, AZ, USA).

### Photo-trapping

Additionally, we performed two campaigns of photo-trapping in the same areas away from farms. In the first site we used nine baited cameras (Cam TrakkerTM 143, Watkinswille, GA, USA) from March to May 2006, while in the second area we used 11 cameras from May to August 2006 (Figure 1).

### Farm surveys

We identified all farms (inhabited and abandoned farms) in the whole study area and evaluated the presence and number of cats during survey visits. The aim of these surveys was to establish the pattern of distribution of cats around farms in the study area, and to describe its relation with farm location and human use. We complemented this information with interviews to the owners/workers to obtain information on their use of the farm, the number and type of cats and on their relationship with cats (including food provisioning).

### Environmental and human-related variables

We selected variables aiming at describing the impact of human-related variables associated with the commensality of cats around people and, additionally, those associated with the plasticity of cats when using more natural areas, including vegetation types. We used a ground-validated 1∶5000 orthophoto (year 2005) to build the digital land cover cartography. We considered three land cover classes: human settlements, corresponding to the building areas in farms and other human settlements; natural vegetation, including sclerophylous vegetation, coniferous forests and riparian vegetation, and agro-forestry areas, which consisted in the oak montado-dehesas without understory and olive groves (Table 1). Additionally, we used a digital elevation model (DEM) in raster format (10 m resolution) obtained from a 1∶25 000 vectorial topographic map. We derived slope from the DEM using second-order finite differences, and ranged from 0 to 41°. We digitised roads and rivers from 1∶250 000 maps, corresponding to the length of paved roads and main rivers. Environmental and human-related variables were determined within a circle centred at each farm main building with 1 km radius (Table 1). ArcView 3.2, Spatial Analyst, Patch Analyst and 3D Analyst extensions were the GIS software applications used.

**Table 1:** Description of the variables used in each data analysis.

Variable types	Code	Range or categorical values	Analysis		
			Presence and abundance	Daily movement	Habitat selection
***Environmental features***					
Elevation range	Elev_range	20–210 m	X	X	X
Number of elevation curves crossed	Elevcurves_c	0–68		X	
Mean slope	Slope	1.6–15.3	X		X
Slope range class	Slope_r	1 for slope range 0–13.5; 2 for slope range >13.5			X
Natural vegetation cover	Natveg	0–75%	X	X	X
Number of patches of natural vegetation crossed	Natveg_c	0–4		X	
Agro-forestry area	AGF	25–95%	X	X	X
River length	River_l	0–7174 m	X		
Number of rivers crossed	Riv_c	0–3		X	
Presence of other carnivores*					
Red fox	VV	0–88%		X	
Badger	MM	0–95%		X	
Stone marten	MF	0–95%		X	
Common genet	GG	0–95%		X	
Egyptian mongoose	HI	58–83%		X	
All species combined	carn	65–95%		X	
***Human features***					
Human settlements area	House	1.1–34	X	X	X
Number of human settlements crossed	House_c	0–2		X	
Human presence	People	0 for absent,1 for present	X		
Minimum distance to human settlements	MDH	147–3373 m	X		
Minimun distance to human settlements with cats	MDCH	245–6630 m	X		
Number of roads crossed	Roads_c	0–3		X	
Length of roads	Road_l	0–3916 m	X		
Distance to nearest road	Road_d	1 for distance range <200 m; 2 for distance >200 m			X
Feeding domestic cats	Cats_feed	0–1	X		

*Estimated as the average of Kernel isolines crossed.

Variables used in **presence and abundance** analyses refer to a 1 km radius around houses, in movement analyses they refer to an Lm band along the movement vector (length of the **daily movement**), and in **habitat selection** they are calculated on home ranges.

### Analysis of the presence and the abundance of cats at farms

We analysed the environmental and human-related factors affecting the presence of cats at farms using generalised linear models with a binomial error distribution and a logit link function (glm function in R Software version 9.1). We considered that two independent variables were strongly correlated when r_s_>0.7, selecting the one with a higher correlation with the dependent variable and/or the variable with most clear biological interpretation [36]. In total, we generated 17 a priori models of cat presence at farms based on three groups of hypotheses depending on the combination of variables: 1) human influence; 2) environmental variables; or 3) a combination of both (Table 2). In the case of cat abundance, we used a Poisson distribution with a log link. Following the same procedure, we generated 16 candidate models (Tables 1 and 2). We used the Akaike Information Criterion (AIC) to rank the models according to their capacity to describe the data parsimoniously [37].

**Table 2:** Summary of best models describing domestic cat presence and abundance (GLM) in rural farms and their daily movements (GLMM).

Models	Model code	Deviance	AIC	*w*AIC
Presence of domestic cats in farms				
Intercept only	A0	157.4	159.4	-
People+Road_l+River_l	A8	109.4	117.4	0.28
People+Road_l	A17	111.7	117.7	0.23
People+Road_l+River_l+Elev_range	A7	108.2	118.2	0.18
People+Road_l+MDH	A16	111.4	119.4	0.10
People+Road_l+River_l+Elev_range+MDH	A6	107.7	119.7	0.09
Abundance of domestic cats in farms				
Intercept only	B0	34.9	150.6	-
cats_feed+People+Slope	B8	20.2	141.9	0.25
cats_feed+People	B16	22.7	142.3	0.20
cats_feed+People+MDH	B15	21.3	143.0	0.15
cats_feed+People+Slope+HOUSE	B7	19.5	143.1	0.14
cats_feed+People+Slope+MDH+HOUSE	B6	18.6	144.2	0.08
cats_feed+People+MDH+Road_l	B14	20.6	144.2	0.08
Daily movements				
Intercept only	C0	1973	1977	-
VV+Elev_range+Roads_c+Riv_c+Natveg _c	C14	484.5	498.5	0.19
VV+Elev_range+Roads_c+Riv_c+Natveg _c+AGF	C13	482.8	498.8	0.16
VV+Elev_range+Roads_c+Riv_c+Natveg _c+season	C6	478.8	498.8	0.16
VV+Elev_range+Roads_c+Riv_c+Natveg _c+AGF+season	C5	477.4	499.0	0.15
VV+Elev_range+Roads_c+Riv_c+Natveg _c+AGF+season+House	C4	476.0	500.0	0.10
VV+Elev_range+Roads_c+Riv_c+Natveg _c+AGF+House	C12	481.8	499.8	0.10
VV+Elev_range+Roads_c+Riv_c+Natveg _c+AGF+House+AGF_c	C11	481.0	501.0	0.05

We run a total of 17, 16 and 22 models, respectively. AIC Akaike Information Criterion, *w*AIC Akaike weights. See Table 1 for a description of the variables.

### Home range analysis

We located the radio marked cats by triangulation using two bearings taken at less than 10 minutes apart to minimize the errors caused by animal movements. Only bearings between 60° and 120° were accepted [38]. We calculated the fixes with 95% error ellipses using length maximum likelihood estimators in LOCATE. We evaluated the location error (∼64 m) during trials when the cats were in known locations inside the farms. The animals were located on average 2–3 times per day at any time in the 24 h. We estimated the home range utilization distribution of the radio-marked animals using a kernel estimator (*kernelUD* function, *adehabitat* package in R software) [39]. The utilization distribution is the bivariate function giving the probability density that an animal is found at a given point according to its geographical coordinates. Using this model, we defined the home range as the minimum area in which an animal has some specified probability of being found [38], [40]. We estimated the individual home ranges with the 95% utilization distributions.

### Habitat analysis

We investigated cat habitat use using five covariates, which expand in habitat categories (Table 1). We compared between the habitat used and the habitat available within their home ranges [40]. First, we used a compositional analysis to obtain a rank order of preferences, testing the overall significance of the selection with a Wilks lambda and then building a ranking matrix [40]. Ranking matrices for domestic cats compare proportional radio-locations for each individual in each habitat type with the proportion of each habitat type available within the home ranges. Additionally we used the Eigen analysis of selection ratios and the graphical approach to describe habitat selection (using the adehabitat package in the R software v.9.1) [41]. This method undertakes an additive linear partitioning aiming at maximizing the difference between habitat use and availability in the first factorial axes. The habitat types with a selection ratio between 0 and 1 are used below their availability while those above 1 are positively selected.

### Daily movement analyses

We analysed the environmental and human-related factors affecting the length of the movements of the radio-tracked cats. These variables represent the most important environmental and human features related with the behaviour and ecology of domestic cats [3], [12], [15], [42]–[45]. Additionally, we included several variables describing the probability of encountering other carnivores during the displacement (Table 1). With this purpose we performed 54 transects of 1 km in which we surveyed signs of carnivore presence within the minimum convex polygon defined by all the locations of the marked cats. Transects were located evenly in areas in which we had entrance granted by landowners, and following dirt roads or foot-paths facilitating the surveys. We built a kernel utilization distribution for each species using all the signs of presence detected and an additional one for all the species combined. We used the number of kernel probability isolines (in 5% increments) crossed by cat displacements as a proxy of potential interference between wild carnivores and domestic cats (Table 1). We built a set of displacement vectors using two consecutive locations as close as possible to 24 hours (ie, each vector was defined by two locations obtained in consecutive days). Each displacement was divided by location error (64 m) in order to standardize the length size (spatial resolution) and avoid over dispersion. In this way, the analysis will show mostly the determinants of longer displacements. We used generalised linear mixed models using the length of the daily displacement as response variable and a group of independent variables: season (fixed factor), sex (fixed factor), % of natural vegetation cover in each segment (Natveg), number of patches of natural vegetation crossed (Natveg_c), % of agro-forestry area (AGF), number of agro-forestry patches crossed (AGF_c), number of rivers crossed (Riv_c), average of kernel probability levels of carnivore occurrence crosses (e.g. VV), number of roads crossed (Roads_c), human settlements area (House), number of human settlements crossed (House_c), elevation range (Elev_range) and number of elevation curves crossed (Elevcurves_c) (Tables 1 and 2). The variables quantifying the percentage of land use classes along the displacements were built using a 5 m buffer around each displacement vector (Table 1). Again, we removed one independent variable when it showed a strong correlation with other one, retaining the one with the higher correlation with the dependent variable. We used a code identifying each individual as a random factor in all models, obtaining 22 a priori models according to the potential factors that could affect the displacements (Table 2). We used the lme4 package in **R** software v.2.9.1 [46].

## Results

### Domestic cat occurrence: live- and photo-trapping

In spite of our efforts, we were unable to detect any domestic cat in the two trapping sites selected in the natural area away from farms (Figure 1). At the northern trapping site, with an effort of 1464 trap-nights we live-captured 7 common genets (*Genetta genetta*), 7 red foxes (*Vulpes vulpe*s), 3 Egyptian mongooses (*Herpestes ichneumon*), 3 badgers (*Meles meles*) and 2 stone martens (*Martes foina*). At the same site, we obtained 498 photos with an effort of 612 trap-nights. Considering only one capture per day and camera we photo-captured 15 wildboars (*Sus scrofa*), 13 red foxes, 8 badgers, 8 common genets, 7 Egyptian mongooses and 4 stone martens. Similarly, at the Southern site, with an effort of 1117 trap-nights, we live captured 12 red foxes, 5 Egyptian mongooses, 3 badgers, 2 common genets, and 2 stone martens. Photo-trapping provided 480 photos with an effort of 814 trap-nights, including 22 red deer (*Cervus elaphus*), 8 wildboars, 9 red foxes, 8 common genets, 7 Egyptian mongooses, 5 badgers, and 2 stone martens.

On the contrary, at the ten farms where we carried out a trapping effort of 297 trap nights we captured 30 different individuals: 12 males (of which 8 were adults) and 18 females (12 adults). After these results we concentrated our work in and around farm buildings and in the adjacent scrubland areas.

### Cat presence and abundance at farms

We identified 128 farms within our study area, the majority of which (67.2%) had no resident people. Many of them were abandoned or even in ruins. There were no cats in the abandoned houses. The average distance to the nearest house was 1.1±5.51 km (±SD). The 42 farms in use give a density of 0.09 farms km^−2^. Cats were present in 39 of them (92.5%), with a total of 130 individuals (3.3±1.85 cats per farm) and a density of 0.26 cats km^−2^ (this density excludes the rural area of the village of Barrancos and a buffer of 3 km around). The sex ratio of 88 individuals (29 males, 59 females) was 1M∶2F but for the remaining 41 individuals sex was unknown. The average nearest distance between farms with cats was 2.7±12.7 km. Farm owners or users considered all cats to be free ranging i.e., they were no kept as pets. In none of the farms the cats received veterinary support. Food provisioning was never provided on a regular basis, with 33 farms feeding cats only sporadically with human refuse (84.6% of the occupied houses). Except for one cat out of the 130, farm owners defined them as very wary (cannot be captured by hand, fleeing when approached).

The best model describing the presence of domestic cats in farms included the presence of people and the length of roads and rivers around the farm (explaining 30.5% of the deviance); the next model included only the first two variables (models A8 and A17, Table 2). The most important predictor of cat presence was the occupation of farms by people, accounting for 90.6% of the deviance explained by the best model (Table 3). The other two predictors are also associated to the farms that are more intensively used by people, either because they are better communicated (road length) or because the area is more suitable for small-scale traditional agriculture (river length). These results show that the presence of cats in natural sites far from urban, suburban and rural areas still rely heavily on human-related variables.

**Table 3:** Standardized parameter estimates for the variables included in the models with the highest support (higher *w*AIC in Table 2) for presence, abundance and movement of domestic cats.

Models/variables	Standardized Estimate	S.E.	*Z*	*P*
Presence of domestic cats in farms				
Intercept	−1.15	0.26	−4.41	<0.0001
People	1.32	0.23	5.75	<0.0001
Road_l	0.40	0.24	1.72	0.0857
River_l	0.37	0.25	1.49	0.1358
Abundance of domestic cats in farms				
Intercept	1.16	0.10	12.20	0.5355
Cats_feed	0.27	0.13	2.18	0.0291
People	0.25	0.11	2.38	0.0174
Slope	−0.15	0.10	−1.53	0.1258
Daily movements				
Intercept	1.63	0.13	12.38	<0.0001
VV	−0.58	0.04	13.54	<0.0001
Elev_range	0.28	0.03	10.76	<0.0001
Roads_c	0.14	0.02	8.98	<0.0001
Riv_c	0.14	0.02	7.97	<0.0001
Natveg_c	0.04	0.02	1.88	0.0596

SE is the standard error, ***Z*** value is the Wald statistic and ***P*** is the significance. See Table 1 for a description of the variables.

In the analysis of the variables that influence the number of domestic cats per farm all models with the highest support included food supplementation by people and the presence of people (Table 2). The most supported model (B8, Table 2) also included the mean slope around the farm, which had a negative effect (Table 3). Together the two human related variables explain 83.2% of model deviance, with partial contributions of 45.1% and 38.2%, respectively for *cats_feed* and *people*.

### Home ranges

We marked eight cats (5 males, 3 adults and 2 subadults; 3 adult females) with radio-collars. On average, we tracked them for 10±0.7 (±SD) months, obtaining an average of 176±14.8 fixes per animal. The area covered by all the radiolocations of the marked cats was 10,416 ha (calculated as the minimum convex polygon). All individual home ranges included the farm where each cat was captured (Figure 1). The average of the maximum distance between the capture site (farm) and the furthest radio location was 2.9±1.8 km, ranging between 1.2 and 6.3 km. As expected, male home range sizes were larger than those of females, with 430 ha (range 71–1,476) and 87 ha (41–113), respectively. There was a substantial inter-sexual home range overlap (Figure 1). Home ranges were centred in farm buildings, but in some occasions males moved away to another farm or to the village. In fact, the furthest distances away from the farm belong to males during the mating season (Autumn-Winter), on average 3.8±2.2 km (with a maximum of 6.4 km) *vs* 1.6±0.7 km out of the mating season; for females the average furthest distance was 1.2 for both seasons (±0.4 and ±0.3, respectively). The maximum distances that males travelled are associated to the distance to the nearest farm with cats (*r* ^2^ = 0.67) only during the mating season (*r* ^2^ = 0 outside the season). Males used farms as stepping stones in their mating displacements; the maximum inter-farm distance moved was 2.2 km while the male that never made any excursion to other farm was 3.4 km away from the nearest occupied farm. None of the females moved between farms.

### Habitat use

**Figure 2:**
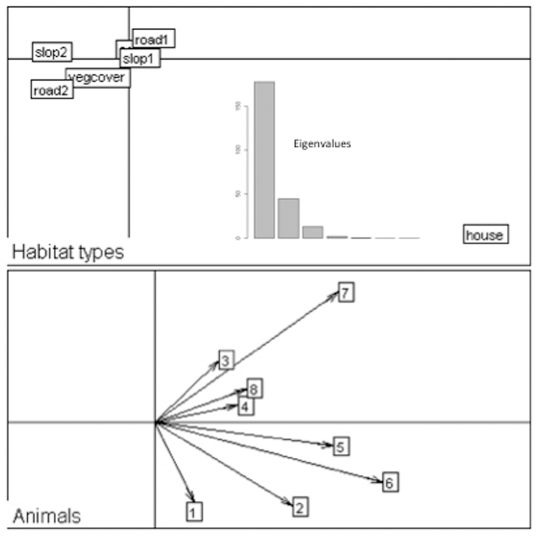
Eigen analysis of selection ratios of habitat selection by the relocations of domestic cats in the seven habitat types within their home ranges [**41**]. Top: Habitat type loadings on the first two factorial axes. The cross shows the position of a hypothetical habitat type unused by all individuals. Bottom: Individuals scores on the first factorial plane. The numbers corresponds to the animals: 1- F060, 2 – F178, 3 – F290, 4 - F240, 5 – F310, 6 – F230, 7 – F360, 8 – F200.

The radio-tracked cats did not use the different land-use types available within their home ranges at random (λ = 0.0122, *P* = 0.008). The compositional analysis showed that there was a clear order of preference headed by human settlements (*House*), followed by areas at less than 200 m from roads and with a smaller slope (Table 4). Steep areas, far from roads and covered with natural vegetation were the less preferred (Table 4). The Eigen analysis of selection ratios confirmed those clear preferences. The results for the first two axes explain 93.3% of the information (74.4% for the first axis and 18.1% for the second, Figure 2). *House* was the land-use type more used by cats; in fact, seven of the eight individuals made it their first choice as shown by the highest selection ratios (Table 4). The selection ratios for the remaining habitats show that human settlements play an important role because cats spent most of their time in the areas around the houses, i.e., close to roads and with low slope (Table 4).

**Table 4:** Ranking matrices obtained from the compositional analysis and the habitat selection ratios.

	Compositional analysis								Habitat Selection ratios	
Habitat type (use)	Habitat type (availability)							Rank		
	House	Road_d<200	Slope_r<13.5	AGF	NatVeg	Road_d>200	Slope_r>13.5		average	SE
House		+++	+++	+++	+++	+++	+++	1	3.19	0.56
Road_d<200	−−−		+	+	+++	+++	+++	2	1.14	0.07
Slope_r<13.5	−−−	−		+	+++	+++	+++	3	1.07	0.02
AGF	−−−	−	−		+	+++	+++	4	1.02	0.03
NatVeg	−−−	−−−	−−−	−		+++	+++	5	0.82	0.09
Road_d>200	−−−	−−−	−−−	−−−	−−−		+	6	0.57	0.13
Slope_r>13.5	−−−	−−−	−−−	−−−	−−−	-		7	0.40	0.11

In the compositional analysis a positive (negative) sign in pairs of habitats marks the preference between them. A triplet sign represents a positive or negative significant deviation from random at *P* = 0.05, for 500 randomisation tests. The habitat type used less than its availability is characterized by a selection ratio ranging from 0 to 1. The habitat type used more frequently despite their lower availability in the area is characterized by a selection ratio ranging from 1 to infinity [39], [40]. Average is for the value of each individual.

### Daily movements

We obtained 339 daily displacements with a mean length of 605±743 m, and an average time span of 20±8 hours. The results of the linear mixed model showed that sex did not affect the daily displacements and that season only appeared in 3 of the 7 models with *w*AIC>0.05 (Table 2). Environmental variables seem to be very relevant in explaining the length of the daily displacements. In fact, the most supported model includes the average number of red fox 5% kernel isolines crossed, the elevation range, the number of roads and rivers crossed and the number of patches of natural vegetation crossed. The most important variable in all models was the proxy for red fox encounter probability, having a strong negative effect on daily displacement (Table 3). The positive effects of elevation range and number of roads and rivers crossed represents the differences in elevation when the animals moved far away from farms, since the households where we marked the cats are located at elevated places, and the further they moved away, the more probable it was that they crossed a road or a river.

## Discussion

We were unable to detect cats living freely far away from people. Our trapping effort was large enough to assume that in our natural area there were no cats living independently of people (or they were present in very low numbers). Considering the management of problematic of cats in natural areas, the control of populations should be more effective when trapping near human settlements (e.g. our farms). The presence and number of cats was dependent on the presence of people and the resources they provide. In fact, the area around farms was the preferred land use type for cats, as confirmed by the highest value of selection ratios.

Outside islands and Australian mainland domestic cats can become feral not only in rural environments, but also in semi natural environments that are settled by people, as shown in the literature, with no evidence for cats living on their own in natural areas away from people. For example, in Scotland, the putative wildcats were in contact with farm cats [42]; in a natural area of Hungary the marked feral cats were close to farms, and their home ranges were at less than 2 km from one city and a village [15]; in northern France, domestic cats centred their home ranges in a village or around farms [43]. Studies on feral cats are commonly located in urban and suburban areas [e.g. 44], [47] and even in inhabited small islands feral cats tend to rely on people [48]. It is therefore clear that high human density supports higher cat densities in natural or semi-natural areas, linking the expansion success of cats to different levels of human occupation.

Nevertheless, we can still find domestic cats roaming in natural areas far away from any human settlement. In our study area, their home ranges are centred on farms, but males can make long displacements in search of females during the mating season [see also 43], [44]. In fact, female distribution and density is the primary factor determining male range size [12], as demonstrated by the relation between the maximum distance travelled in a season and the distance to the nearest farm with females. The furthest excursion by a male was 6 km away, but in this case, the cat was using several farms as stepping-stones. There seems to be a 3 km distance threshold between farms above which males cannot connect them. Other author [43] detected one male mating excursion between farms separated by 2.5 km. Our study provides a clear linkage between the distribution, numbers and movements of domestic cats and several human and environmental factors, which can be managed to reduce the pressure of this species into natural areas. The presence of people is the first most important variable to be managed. The existence of small settlements or even isolated houses or farms represents a bridge allowing the intrusion of cats into the surrounding areas. Clearly, the planning of new urban areas and the spread of houses and small urban settlements into natural areas should consider the area of influence where we can expect to have an impact from domestic cats. The distance between houses is a key element since cats use them as stepping-stones when moving, even when residents do not own pet cats and do not directly provide food.

In summary, in natural areas cats may live strictly depending on only wild resources, as in some deserted islands, but the general pattern is that they do not. Feral cats have the capacity to move long distances away from households, but still restricting their movements to the vicinity of human settlements. The free ranging domestic dogs *Canis familiaris* have the same behaviour in natural areas with a healthy large carnivore comunity, where their movements were restricted to the vicinity of human dwellings [49], [50]. The success of colonization and population increase of domestic cats in non-native environments is facilitated by the availability and quality of the resources, few natural enemies and the advantageous physical characteristics of the environment [51].

Like in islands, cats living in farms do not compete with other carnivores, but unlike the confined environment of islands, in many mainland areas they have to share space with other predators when moving away from houses. In fact, mesopredator species, like the domestic cat, appear to be ecologically released by increased urbanization not only because they can adapt well to those environments, but also because such sites may provide refuge from top predators [52]. The daily movements of domestic cats show that they strongly avoided entering the areas with higher red fox density. In a study in New South Wales, after fox removal domestic cats showed a significant resource shift, suggesting a strong interspecific competition mediated by both exploitation and interference [45]. Foxes prey on cats and their kittens and cat remains have also been observed in fox diet samples in Europe and Australia [45], [53]–[55]. During our study an Egyptian mongoose predated all kittens of a domestic cat litter in Noudar Castle. Our interpretation is that the presence and abundance of competing predators mediate the differences in presence, abundance and movements of cats in natural areas of islands and mainland.

### Domestic cat management in natural-rural areas

If we cannot manage the presence of people living in the field, in order to maintain low cat numbers, food provisioning should be banned while the access of cats to human refuse must be controlled. In the worst case scenario in which people is living in a network of well-connected settlements and provisioning cats with food, males should be neuter to reduce the distances they move away from houses. Moreover, the movements of domestic cats would be limited in areas with large patches of natural vegetation, promoting the presence of other carnivors such as foxes. Finally, because private landowners are the ultimate controllers of their land, providing them with information is essential to increase the awareness of people before the implementation of any measure. The presence and tolerance of domestic cats in human settlements in rural areas is associated to the ancestral role of cats in controlling rodent populations, but this function can be performed by the barn owl (*Tyto alba*) that also leverages on buildings within human settlements.
